# Etiopathology of chronic tubular, glomerular and renovascular nephropathies: Clinical implications

**DOI:** 10.1186/1479-5876-9-13

**Published:** 2011-01-20

**Authors:** José M López-Novoa, Ana B Rodríguez-Peña, Alberto Ortiz, Carlos Martínez-Salgado, Francisco J López Hernández

**Affiliations:** 1 Instituto de Estudios de Ciencias de la Salud de Castilla y León (IECSCYL), Soria, Spain; 2 Unidad de Investigación, Hospital Universitario de Salamanca, Salamanca, Spain; 3 Unidad de Fisiopatología Renal y Cardiovascular. Departamento de Fisiología y Farmacología, Universidad de Salamanca, Spain; 4 National Institutes of Health, Bethesda MD, USA; 5 Renal and Vascular Research Laboratory, IIS-Fundación Jiménez Díaz and Universidad Autonoma de Madrid, Madrid, Spain; 6 Instituto Reina Sofía de Investigación Nefrológica, Fundación Íñigo Álvarez de Toledo, Madrid, Spain

## Abstract

Chronic kidney disease (CKD) comprises a group of pathologies in which the renal excretory function is chronically compromised. Most, but not all, forms of CKD are progressive and irreversible, pathological syndromes that start silently (i.e. no functional alterations are evident), continue through renal dysfunction and ends up in renal failure. At this point, kidney transplant or dialysis (renal replacement therapy, RRT) becomes necessary to prevent death derived from the inability of the kidneys to cleanse the blood and achieve hydroelectrolytic balance. Worldwide, nearly 1.5 million people need RRT, and the incidence of CKD has increased significantly over the last decades. Diabetes and hypertension are among the leading causes of end stage renal disease, although autoimmunity, renal atherosclerosis, certain infections, drugs and toxins, obstruction of the urinary tract, genetic alterations, and other insults may initiate the disease by damaging the glomerular, tubular, vascular or interstitial compartments of the kidneys. In all cases, CKD eventually compromises all these structures and gives rise to a similar phenotype regardless of etiology. This review describes with an integrative approach the pathophysiological process of tubulointerstitial, glomerular and renovascular diseases, and makes emphasis on the key cellular and molecular events involved. It further analyses the key mechanisms leading to a merging phenotype and pathophysiological scenario as etiologically distinct diseases progress. Finally clinical implications and future experimental and therapeutic perspectives are discussed.

## Introduction to chronic kidney disease

### Definition and clinical course

Chronic kidney disease (CKD) comprises a group of pathologies in which the renal excretory function is chronically compromised, mainly resulting from damage to renal structures. Most, but not all, forms of CKD are irreversible and progressive. Renal damage includes (i) nephron loss due to glomerular or tubule cell deletion, (ii) fibrosis affecting both the glomeruli and the tubules, and (iii) renal vasculature alterations. CKD results from a variety of causes such as diabetes, hypertension, nephritis, inflammatory and infiltrative diseases, renal and systemic infections (e.g. streptococcal infections, bacterial endocarditis, human immunodeficiency virus -HIV-, hepatitis B and C, etc.), polycystic kidney disease, autoimmune diseases (e.g. systemic lupus erythematosus), renal hypoxia, trauma, nephrolithiasis and obstruction of the lower urinary ways, chemical toxicity and others. In a variable number of cases, renal injury by any of these causes evolves towards a chronic, progressive and irreversible stage of increasing damage and renal dysfunction wherein, eventually, renal replacement therapy (RRT, namely dialysis or renal transplant) becomes necessary [1,2].

**Figure 1:**
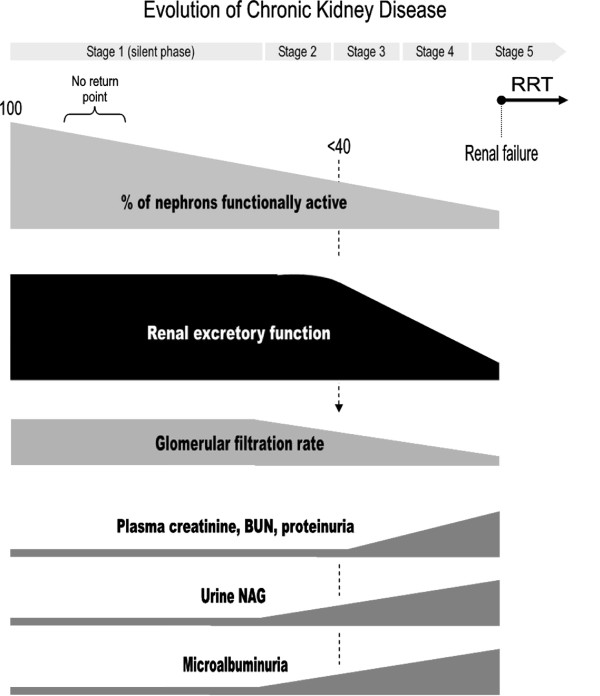
**Graphic representation of the evolution of key pathological events, such as percentage of nephrons functionally active, overall renal excretory function and glomerular filtration rate, and plasma and urine markers associated with time course of chronic kidney disease**. The figure shows the relative priority of appearance of these elements with repect to one another as it occurs in most cases of chronic kidney diseases. Their appearance, however, may vary from this general prototype in specific diseases or in determined cases. In the same way, the slope of increase or decline may also vary. RRT: renal replacement therapy, BUN: blood urea nitrogen, NAG: N-acetyl-β-D-glucosaminidase.

Whether started as glomerular, tubular or renovascular damage, chronic progression eventually converges into common renal histological and functional alterations affecting most renal structures, which lead to progressive and generalized fibrosis and glomerulosclerosis. Once initiated, kidney injury gradually aggravates even in the absence of the triggering insult. Congruently with a common chronic phenotype, CKD can be diagnosed independently from the knowledge of its cause. The National Kidney Foundation (NKF) of the United States of America classifies CKD progression in five stages according to the extent of renal dysfunction and renal damage, symptomatology and therapeutic guidelines (table 1). Late stage 4 and, especially, stage 5 (renal failure) pose a heavy human, social and economic burden [3-6]. Figure 1 depicts the time course of key pathological events [i.e. percentage of nephrons functionally active, overall renal excretory function and glomerular filtration rate (GFR)] and plasma and urine markers, as they appear through the different stages of CKD.

**Table 1:** Stages of chronic renal disease defined by the National Kidney Foundation of the U.S.A. according to the glomerular filtration rate (GFR, in mL/min per 1.73 m^2 ^of body surface), and common manifestations observed in each stage.

Stage	GFR	Common symptoms
1	≥ 90*	**-**

2	60-90*	↑ Parathyroid hormone, ↓renal calcium reabsorption

3	30-59	Left ventricular hypertrophy, anemia secondary to erythropoietin deficiency

4	15-29	↑ Serum triglycerides, hyperphosphatemia, hyperkalemia, metabolic acidosis, fatigue, nausea, anorexia, bone pain

5	< 15	Renal failure: severe uremic symptoms

*CKD is defined as either GFR < 60 mL/min/1.73 m^2 ^for 3 months or a GFR above those values in the presence of evidence of kidney damage such as abnormalities in blood or urine (e.g proteinuria) tests or imaging studies. ↑: increase; ↓: decrease.

The term uremia or uremic syndrome refers to the clinical manifestations of CKD, which are derived from the inability of the kidneys to properly clear the blood of waste products. As a consequence, toxic substances usually eliminated through the urine become concentrated in the blood and cause progressive dysfunction of many (virtually all) other tissues and organs, seriously compromising well-being, quality of life and survival. For example, elevated serum uric acid is a marker for decreased renal function, may have a mechanistic role in the incidence and progression of renal functional decline [7,8]. In a recent study performed on 900 healthy normotensive, adult blood donors higher serum uric acid levels were highly significantly associated with a greater likelihood of reduced glomerular filtration [9]. Further clinical trials are needed to determine if uric acid lowering therapy will be effective in preventing CKD. However, kidney damage must occur to a significant extent before function becomes altered. Uremic signs and symptoms start to be vaguely detectable when at least two thirds of the total number of nephrons is functionally lost. Until then, CKD runs apparently silent. This is due to the ability of the remaining nephrons to undergo hypertrophy and functionally compensate for those that are lost [10].

A representation of GFR evolution in time is a helpful estimation of renal disease progression rate. It is useful to monitor CKD as well as to predict the time for RRT. Progression rate is highly dependent on the underlying cause but, due to genetic heterogeneity, it is also very variable among subjects with the same etiology [2]. In general, tubulointerstitial diseases progress more slowly than glomerular ones, and also than diabetic kidney disease, hypertension-associated disease and polycystic kidney disease. A complete diagnosis includes detection, determination of stage of disease, assessment of etiology, presence of comorbid conditions and estimation of progression rate [3-6].

The key and yet unmet issue in CKD is why, and through which mechanisms, persistence of triggering damage or repetitive bouts, initially repairable as in acute damage events, eventually go beyond a no return point, after which non reversible chronicity ensues. The responses to these questions are beyond our present knowledge of CKD pathology. The development of early diagnostic and prognosis markers, and effective, curative -not merely palliative or delaying- therapies critically depend on our finding answers to these largely ignored questions. Notwithstanding, knowledge has emerged in the last few decades on new mechanisms and molecular pathways that mediate the development of certain facets of chronic phenotypes. This knowledge is potentially useful for optimizing current therapies and for developing new ones. The purpose of this review is to describe the pathophysiological processes leading to tubular, interstitial, glomerular and renovascular chronic diseases, focused on the cellular and molecular mechanisms involved, making emphasis in those that are common for most CKDs regardless of aetiology.

### Etiopathogenesis

A variety of renal injuries may eventually evolve to CKD [2]. Disease may start in the tubules and interstitium (tubulointerstitial diseases), in the glomeruli (glomerular diseases) or even in the renal vascular tree (renovascular diseases), as a consequence of (i) systemic diseases such as diabetes and hypertension, (ii) autoimmune reactions and renal transplant rejection, (iii) the action of drugs, toxins and metals, (iv) infections, (v) mechanical damage, (vi) ischemia, (vii) obstruction of the urinary tract, (viii) primary genetic alterations, and (ix) undetermined causes (idiopathic). Yet, a number of conditions, like genetic cystic diseases, affect renal structures and function through mostly unspecific mechanisms, and evolve into CKD for undetermined reasons.

Some decades ago, the leading cause of CKD was glomerulonephritis secondary to infections. Antibiotics and improved sanitary conditions have laid the way to diabetes and hypertension as the first and second leading causes of end stage renal disease (ESRD) in the developed world, respectively [11]. In fact, about 50% of ESRD patients (in the USA) are diabetic [12]. According to this source, about 50-60% of all patients with CKD are hypertensive, and this figure increases to 90% in patients over 65 years. In the corresponding general population the incidence of hypertension is 11-13% and 50%, respectively. Alltogether, 70% of ESRD cases are due to diabetes and hypertension [13]. Recently, several large-scale epidemiological studies [14-16] have identified obesity as an independent risk factor for CKD. The link between obesity and CKD is not fully explained by the association between obesity and diabetes or hypertension respectively [17]. Hall et al. [18] described a progressive increase in the incidence of ESRD since the eighties, coinciding with an increase in obesity and decreased hypertension. Similarly, Chen et al. [19] showed an association between the metabolic syndrome and the risk of developing chronic renal failure. Both studies support the association between increased weight and kidney disease, although no direct causality link between obesity and CKD can yet be established [20].

### Genetic predisposition

A genetic predisposition for renal failure is demonstrated by the 3-9 times higher probability of ESRD in patients with a family history of CKD, compared to the general population [21]. However, it is difficult to assess whether this predisposition is due to a specific susceptibility to undergo renal damage, or to other comorbid conditions generally accepted to have poly- or oligo-genetic components, like hypertension, diabetes or atherosclerosis. Still, this observation has launched the search for nephropathy susceptibility genes.

Except for monogenic diseases (e.g. polycystic renal disease) [22], genetic studies based on quantitative trait loci (QTLs) analysis and sub-pair analysis have been unable to demonstrate polymorphism associations valid for most forms of CKD. A number of polygenic minor gene-gene interactions have been associated with specific human CKD of different etiology, such as type 2 diabetic nephropathy [23]. Several loci have been identified on chromosome 3q, 10q and 18q for diabetic nephropathies, and on 10q also for non-diabetic nephropathies [24]. Recently MYH9 gene polymorphisms have been shown to account for much of the excess risk of HIV-associated nephropathy, hypertensive, diabetic and nondiabetic kidney disease in African Americans [25-27]. A number of mutations have been associated to focal and segmental glomerulosclerosis during the last decade including: (i) two polimorphisms of apolipoprotein L 1 (APOL1) have been associated to the disease in African descendents [28]; and (ii) genetic alterations in five proteins expressed in podocytes, namely podocin (NPHS2 gene) [29,30], inverted formin (INF2 gene) [31], the transient receptor potential cation channel, subfamily C, member 6 (TRPC6 gene) [32], CD2 associated protein (CD2AP gene) [32], and alpha-actinin 4 (ACTN4 gene) [32].

Genetic analysis of renal damage-prone rats crossed with more resistant strains have revealed the existence of 15 loci associated with renal disease [33], three of which coincide with those found in human monogenic segmental glomerulosclerosis, Pima Indians kidney disease, and creatinine clearance impairment in African- and Caucasian-Americans [34,35]. These studies highlight the potential predictive value of animal models for the identification of CKD-associated genes. Still, other genetic determinants present in humans and absent in most animal models, derived from the inter-race, inter-population and inter-individual genomic heterogeneity, may pose limitations to findings make in animals. For example, human leukocyte antigen (HLA)-dependency of renal disease prevalence has been demonstrated in several studies with human populations surveyed for e.g. diabetic nephropathy [36,37] or membranous glomerulonephritis [38].

## Tubular diseases

The terms tubular diseases, tubulointerstitial diseases, tubulointerstitial nephritis and tubulointerstitial nephropathies refer to a heterogeneous panel of alterations which primarily affect both cortical and medullary tubules and the interstitium, and secondarily other renal structures such as the glomeruli [39]. Tubules are the main component of the renal parenchyma and receive the most part of injury in renal disease [39]. Nevertheless, renal interstitium also plays an important role in tubulointerstitial nephropathies, since pathogenesis perpetuates in this compartment and interstitial alterations contribute to diminish renal function [40]. The interstitium is formed by the intercellular scaffolding posed by the extracellular matrix (ECM) and basement membranes, in which several cell types can be found. Apart from those forming blood and lymphatic vessels, including microvascular pericytes, resident and infiltrated immune system cells can also be found (i.e. white blood cells including macrophages). Finally, fibroblasts and, especially under pathological conditions, myofibroblasts form part of the tubular interstitium. Primary tubulointerstitial diseases [41] are idiopathic, genetic or due to (i) the chemical action of toxics and drugs that accumulate in the tubules inducing apoptosis or necrosis of tubular epithelial cells; (ii) infection and inflammation of the tubulointerstitium as a result of reflux/chronic pyelonephritis or other causes; (iii) increased intratubular pressure induced by mechanical stress and related to obstruction of lower urinary tract caused by lithiasis, prostatitis, fibrosis, or retroperitoneal tumors; and (iv) transplant rejection due to immune response. In many cases, the cause of the disease remains unknown. Renal function progressively deteriorates as a consequence of dysfunctional processes of tubular reabsorption and secretion, activation of tubular cells with recruitment of inflammatory mediators, progressive tubule loss and tissue scarring, and eventual damage of other renal structures (e.g. the glomeruli).

Independently of the triggering cause, characteristic hallmarks of tubulointerstitial diseases are tubular atrophy, interstitial fibrosis and cell infiltration [39], resulting in a significant increment in interstitial volume [42,43]. In early stages, glomerular filtration becomes slowly altered, and tubular dysfunction constitutes the main manifestation of tubulointerstitial nephropathies [39,44]. In contrast to glomerular diseases, in tubulointerstitial diseases hypertension appears late and only after a significant fall of GFR [45-47]. Proximal tubule alterations induce bicarbonaturia, β2-microglobulinuria, glucosuria and aminoaciduria. Distal alterations induce tubular acidosis, hyperkalemia and sodium loss [48]. Structural alterations in medulla cause nephrogenic diabetes insipidus that is clinically manifested as polyuria and nocturia [49].

**Figure 2:**
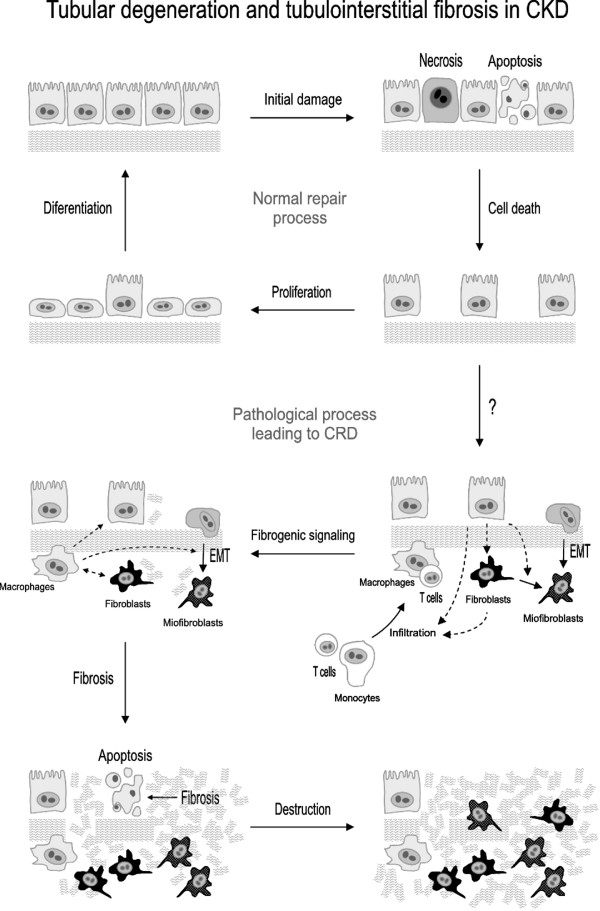
**Schematic depiction of the pathological process of tubular degeneration and tubulointerstitial fibrosis characteristic of tubulointerstitial diseases, and also of later stages of glomerular and renovascular diseases leading to chronic kidney disease (adapted from references **[87]**and **[291]**)**. EMT, epithelial to mesenchymal transition.

Tubulointerstitial diseases can be considered as perpetuating inflammatory responses that escape normal defense and restorative mechanisms [50]. The immune response includes recognition of the insult, an integrative phase and an executioner response. This response is carried out by the complex, integrated and coordinated participation of tubular epithelial, interstitial and infiltrated cells. This process is mediated by chemotactic, proinflammatory, vasoactive, fibrogenic, apoptotic, and growth-stimulating cytokines and autacoids, which are released by participating cells, as well as by overexpression of specific receptors for these molecules, and antigenic and adhesive surface markers expressed in target cells [51-55]. The sequence of pathogenic events during tubulointerstitial fibrosis starts with the initial damage that activates inflammatory and repair mechanisms in the kidneys, and follows with a stage of fibrosis that leads to progressive tissue destruction (figure 2). These events are described in the next sections.

### Initial damage and cell activation

As a consequence of the damage inflicted to tubular structures by the triggering insult, an initially restorative response starts, which eventually corrupts into a pathological vicious cycle of interstitial fibrosis and tissue destruction. Depending on the insult, tubular epithelial cell necrosis, apoptosis, or both are observed. In a restorative effort, an inflammatory response is implemented and tubular cells proliferate to substitute for dead cells. For unknown reasons, under undetermined circumstances the restorative process (in this and the next phases -see below-) loses the appropriate regulation and takes an irreversible self-destructive course that does not need the presence of the initial insult to progress. Interstitial fibrosis results from a deregulated process of fibrogenesis initially required to rebuild the normal tissue structure posed by ECM and basement membranes [56]. Rather early, interstitial fibrosis gains a central pathological role, scars the interstitium and epithelial areas that should have been repaired with new epithelial tubular cells, and induces further tissue damage and destruction through apoptosis and phenotypical transdifferentiation of epithelial tubular cells.

**Figure 3:**
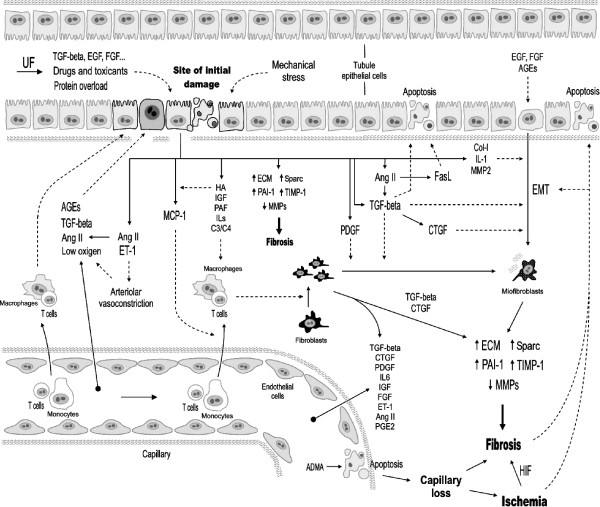
**Extracellular mediators and effectors of tubulointerstitial pathological events in chronic kidney disease**. ADMA: asymmetric dimethylarginine. HA, hyaluronic acid. C3 and C4, factors 3 and 4 of the complement. UF, ultrafiltrate.

Tubular epithelial cells respond to the initial insult by (i) proliferating or (ii) dedifferentiating through an epithelial to mesenchymal transition (EMT)-like process that allows them to migrate, proliferate and eventually redifferentiate [57,58]. EMT from tubule cells to fibroblasts is an undetermined mechanism of fibrosis. It is often recognized as an important contributor to fibrosis [59-61], although this concept has been challenged (see the debate in 62). Evenmore, in the fibrosis observed in the transition from acute kidney injury to CKD, myofibroblast have been shown to be mostly originated from fibroblasts and pericytes and not from tubule epithelial cells [63,64]. As commented above, the skewed repair process gives way to a fibrotic process mediated by activated resident fibroblasts [42], by EMT-derived myofibroblasts [57] and by secretion of (i) cytokines that attract mononuclear cells, (ii) growth factors that stimulate interstitial fibroblasts, and (iii) proinflammatory and profibrotic molecules that stimulate the synthesis of both basement membrane and tubulointerstitial ECM proteins, such as collagens I and IV, fibronectin and laminin [65,66]. Critical events acting on tubular epithelial cells induce the early deposition and accumulation of ECM components in the interstitial compartment. Apical stimulation is exerted on the tubular epithelium by mechanical or chemical action of the glomerular ultrafiltrate, derived from an increased GFR per individual remnant nephron resulting in an increased filtration of proteins, chemokines, lipids and hemoproteins [65]. Basolateral stimulation originates from mononuclear cells and from hypoxia and ischemia resulting from postglomerular capillary loss. Peritubular capillary loss has been demonstrated in animal models of CKD, which has been associated to tubulointerstitial ischemia and fibrosis [67]. It has been suggested that capillary loss is the result of NO synthesis inhibition, because hydrolysis of the endogenous NO synthase inhibitor asymmetric dimethylarginine (ADMA) with exogenous dimethylarginine dimethylaminohydrolase, reduces the extent of capillary loss and renal damage [67]. Indeed, capillary loss is a pathological mechanism associated to CKD progression and nephron loss [68]. A number of mediators are known to participate in these tubular events, which are summarized in table 2 (see also figure 3).

**Table 2:** Main molecular mediators known to participate in the pathophysiological process of tubular degeneration and interstitial fibrosis, grouped according to their most important effect.

ENDOGENOUS ACTIVATORS	ORIGIN	FBR & EMT	INF	TD	ISCH	REFERENCES
**1. Fibrosis and EMT**						
TGF-β	TC, F, MF, P, iG	X				EMT [252,253]; secretion of profibrotic MCP-1 [254] and CTGF [255]. Fibrosis: ↑ECM components and PAI, and ↓MMPs [51,104-106]
EGF	P, UF	X				EMT [256]
FGF	P, UF	X				EMT [234]; fibrosis [87,257-259]
PDGF	P, RC	X				Fibroblast to myofibroblast transformation [87], proliferation of myofibroblasts [260]
CTGF	TC	X		X		EMT, fibrosis, apoptosis [255,261,262]
SPARC	TC, F, MF	X				↓cell adhesion and proliferation, activates TGF-β and collagen I and fibronectin synthesis [98,263]
Thrombospondin	TC, F, MF	X				Activates TGF-β [99]
Decorin and biglycan	TC, F, MF	X				Reservoires of bFGF and TGF-β [101,102].
Collagen I	F, MF, TC	X				EMT [264]
PAI-1	TC, F, MF	X				ECM accumulation and fibrosis [265]
TIMP-1	TC, F, MF	X				Fibrosis ? [87,108]
***2. Inflammation***						
Complement C3 and C4	P, TC	X	X			Inflammation and fibrosis [266-269]
MCP-1	TC, P, iG	X	X			Cell infiltration, fibrosis [72,74,254]
ICAM-1 and VCAM-1	EC, TC		X			On EC: diapedesis and infiltration [270]; On TC: uncertain [271,272]
Hialuronic acid	TC, F, MF		X			Inflammation, MCP-1 and secretion of adhesion molecules [97,98]
***3. Tubular damage***						
Protein overload	UF			X		Tubule cell activation [65] and release of ET-1 [273], ANG-II [274], MCP-1, and RANTES [275]
Complement C5b-9	P	X		X		Tubular damage and fibrosis [276]
TNF-α, IFN-γ, Tweak	iWBC	X	X	X		Inflammation, cell death, fibroblast and myofibroblast activation [277-279]
***4. Ischemia***						
Endothelin-1	TC	X			X	Vasoconstriction and ischemia [273,280]; ↑ECM components and TGF-β [87]
RAS	EC, TC, P				X	Vasoconstriction, ischemia and TGF-β secretion [87,281-284]
ADMA	Plasma	X			X	Vasoconstriction [67]
						
**ENDOGENOUS INHIBITORS**	**ORIGIN**	**FBR & EMT**	**INF**	**TD**	**ISCH**	**REFERENCES**
***1. Fibrosis and EMT***						
Collagen IV	F, MF, TC	X				Inhibits EMT [285]
MMP-2 and 9	TC	X				Degrade collagen IV [286]
HGF	P	X				Inhibits EMT and fibrosis [287-290]
BMP-7	P, TC?	X				Inhibits EMT and fibrosis [285]

ADMA: asymmetric dimethylarginine; EC: endothelial cells; F: fibroblasts; iG: inflamed glomeruli; iWBC: infiltrated white blood cells; MF: myofibroblasts; P: plasma; RC: renal cells (unspecified); TC: tubular cells; UF: glomerular ultrafiltrate.

Infiltrated cells, spanning the endothelium of peritubular capillaries [69], or proliferating resident macrophages [70], essentially contribute to the progression of renal parenchymal damage in CKD [50]. Chemoattractans secreted from the basolateral membrane of damaged tubular cells or crossing the tubule wall from the luminal filtrate, recruit inflammatory cells (monocytes and lymphocytes) and induce fibroblast proliferation. This event, in turn, potentiates a vicious circle of inflammation and fibrogenesis [71]. Specifically, activated tubular cells synthesize the chemoattractant cytokine MCP-1 as a response to protein overload [72]. Tubular MCP-1 production has been documented in patients with CKD [73] and animal models [74]. MCP-1 may also proceed from the proteinuric glomerular ultrafiltrate, originating in plasma or damaged glomeruli. Importantly, MCP-1-deficient mice undergo a milder interstitial inflammation and show a higher life expectancy than controls during CKD [74]. Interstitial accumulation of monocytes and activation of resident macrophages amplify the inflammatory response and lymphocyte diapedesis [69], and contribute to damage progression as sources of profibrotic factors [50].

Damage also activates renal fibroblasts, which proliferate and constitute an important source of pathological, fibrogenic ECM components, such as collagens and fibronectin [42,61,75,76] in response to many factors released from primed tubular cells, white cells and fibroblasts themselves. These molecules include cytokines and growth factors, such as transforming growth factor beta1 (TGF-β1), MCP-1, connective tissue growth factor (CTGF), insulin-like growth factor (IGF), platelet-derived growth factor (PDGF), platelet activating factor (PAF), and interleukins (ILs) 1, 4 and 6, as well as vasoactive molecules (e.g. angiotensin II and endothelin-1), and ECM-cell interaction molecules (e.g. integrins, hialuronic acid) [[65]; table 2; figure 3].

In most forms of CKD, the number of interstitial myofibroblasts is increased, and strongly correlates with the degree of interstitial fibrosis [77,78]. Activated myofibroblasts constitute a predicting histological marker for the progression of renal disease [79,80]. Myofibroblasts are the main source of excessive ECM in fibrotic nephropathies [51]. Myofibroblasts may be originated by trans-differentiation of fibroblasts, tubular epithelial cells, vascular pericytes and macrophages [57,81,82]. In diseased kidneys, myofibroblasts accumulate around damaged tubules and arterioles. Fibrosis-induced microvascular obliteration and vasoconstriction is mediated by vasoactive factors (e.g. angiotensin II and endothelin-1), which produce ischemia, glomerular hemodynamic alterations and further angiotensin II production, all of which amplify fibrogenesis and perpetuate damage [83,84] with the concourse of TGF-β1 and PDGF [85,86].

### Fibrosis

Under pathological conditions during CKDs, damaged renal tissue is replaced by a scar-like formation, characterized by excessive ECM accumulation and progressive renal fibrosis. Fibrosis is the consequence of (i) an increased synthesis and release of matrix proteins from tubular cells, fibroblasts and mostly myofibroblasts, and (ii) a decreased degradation of ECM components [87,88]. During progression of tubulointerstitial fibrosis, fibroblasts show a higher proliferation rate, differentiation to myofibroblasts, and alteration of ECM homeostasis [42]. Although in wound-healing studies it has described an antifibrotic role for macrophages due to their participation in the resolution of the deposited ECM through phagocytosis [89], many short-term studies relate the number of infiltrated macrophages with the extent of fibrosis and kidney dysfunction [reviewed in [90]], supporting an etiological role of these cells in the pathogenesis of renal damage. Moreover, attenuated accumulation of macrophages in experimental obstructive nephropathy is accompanied by enhanced renal interstitial fibrosis and profibrotic activity [91]. However, longer-term studies reveal a reciprocal relationship between these two parameters and raise some questions about the function of infiltrating cells [92]. Thus, probably machrophages play a dual effect, with a short-tem profibrotic effect, and a long-term healing effect.

The interstitial wound in the fibrotic kidney is formed by excessive deposition of constituents of the interstitial matrix (e.g. collagen I, III, V, VII, XV, fibronectin), components restricted to tubular basement membranes in normal conditions (collagen IV and laminin), and *de novo *synthesized proteins (tenascin, certain fibronectin isoforms and laminin chains) [93]. Fibronectin, with chemoattractant and adhesive properties for the recruitment of fibroblasts and the deposition of other ECM components [94], is one of the first ECM proteins to accumulate as a response to the initial damage. Fibroblasts, myofibroblasts, macrophages, mesangial and tubular cells are sources of fibronectin in inflammation and fibrogenesis [95,96]. Other upregulated components in the interstitium of fibrotic kidneys are hialuronic acid [97,98], secreted protein acidic and rich in cysteine (SPARC; 98), thrombospondin [99,100], decorin and biglycan [101,102] (see table 2 and figure 3).

Certain types of CKD are caused by a marked alteration of renal collagenase activity with small or no changes in collagen synthesis. Renal fibrosis in mice with ureteral obstruction is also the result of decreased collagenolytic activity [103]. In damaged kidneys, upregulation of TGF-β activation also contributes to override the natural ECM homeostatic equilibrium by downregulating the expression of determined MMPs and activating the expression of the MMP-inhibitor plasminogen activator inhibitor 1 (PAI-1; 51,104-106). Also TIMP-1, an endogenous tissue inhibitor of MMPs, is actively synthesized by renal cells in progressive CKD [107], and its expression is stimulated by TGF-β, TGF-α, epithelial growth factor (EGF), platelet-derived growth factor (PDGF), tumor necrosis factor alpha (TNF-α), interleukins 1 and -6, oncostatin M, endotoxin, and thrombin [87]. However its role is controversial because TIMP-1 deficient mice show no significant differences in interstitial fibrosis during induced renal damage [87,108].

### Progressive tissue destruction

Tubular atrophy is a histological feature of progressive CKD [109]. Excessive accumulation of ECM, together with expansion and inflammation of the extracellular space, has destructive effects on renal parenchyma and renal function [109]. Loss of tubular cells occurs during the destructive phase as a consequence of apoptosis, persistent EMT (with an undetermined contribution), and interstitial scarring [110]. At this stage, unbalanced fibrogenesis may also contribute to tubular cell death. Interstitial fibrosis impairs oxygen supply to tubular and interstitial cells, which leads or sensitizes to apoptosis [111]. A relevant apoptosis effector in CKD is the Fas-initiated extrinsic pathway [112]. In fact, attenuated expression of the apoptosis-mediated receptor Fas and the endogenous agonist Fas ligand (FasL) reduced tubular epithelial cell apoptosis in an *in vivo *model of diabetic nephropathy [113]. However, in normal circumstances, many epithelial cell types, including renal tubular epithelial cells, are refractory to Fas stimulation-induced apoptosis [114]. Inadequate Fas clustering and altered equilibrium of pro- and anti-apoptotic intracellular modulators may explain the lack of sensitivity to Fas [115,116]. Specifically, signaling at the level of the death-induced signaling complex (DISC) formed around Fas upon receptor stimulation is due to basal expression of Fas-associated death domain-like IL-1-converting enzyme-like inhibitory protein (FLIP), an endogenous inhibitor of DISC [117]. FLIP antisense or cycloheximide treatment, which also drastically reduces cellular levels of FLIP, make refractory fibroblasts to undergo apoptosis upon Fas stimulation. Accordingly, priming stimulation is necessary to make epithelial tubule cells sensitive to Fas-mediated apoptosis, as it occurs in CKD.

TGF-β intervenes in tubule apoptosis *in vivo *as demonstrated by the reduced apoptosis after treatment with an anti TGF-β1 antibody in rats with ureteral obstruction [86-118]. Given its central role in CKD [110], TGF-β poses a good candidate for priming tubular cells to Fas-induced apoptosis. Another candidate for mediating sensitization to Fas-induced apoptosis is angiotensin II. *In vivo*, inhibition of angiotensin II results in a strong amelioration of CKD-associated damage, including tubular epithelial cell apoptosis [119]. *In vitro*, angiotensin II induces apoptosis in rat proximal tubular epithelial cells, and this effect is mediated through the synthesis of TGF-β followed by the transcription of the cell death genes Fas and FasL [120]. In this setting, treatment of tubular epithelial cells with an anti TGF-β neutralizing antibody partially inhibits, while an anti FasL antibody strongly inhibits angiotensin II-induced apoptosis. IL-1 and hypoxia also induce an upregulation of Fas expression in tubule cells [121-123]. Very recently, it has been shown that confined tubular overexpression of TGF-β in mice produces massive proliferation of peritubular cells, widespread fibrosis and focal nephron loss associated to tubular cell dedifferentiation and autophagy [124], although the role of autophagy in tubule cell death needs to be further explored.

The interplay of these and other factors need to be further explored in order to understand the onset of apoptosis in tubular cells during CKD [125]. Furthermore, angiotensin II is a regulator of renal cell function, including tubular cells under physiological conditions [126]. This duality could be related to the fact that cell-to-cell and ECM-to-cell interactions, as well as specific humoral determinants present in different pathophysiological circumstances condition the effect of angiotensin II on cell fate and function. For example, the collagen discoidin domain receptor I is involved in survival of tubular Madin-Darby canine kidney (MDCK) cells [127]. As such, an excessive collagen I and fibronectin deposition may alter cell sensitivity to apoptosis [128]. A number of circumstances must hypothetically be present to let angiotensin II (and other mediators) induce apoptosis *in vivo*, such as a determined humoral coactivating context, and ECM homeostatic disruption caused by fibrogenesis. Probably, persistence of angiotensin II contributes to generate these permissive phenotypes. Finally, ischemia may also directly induce or sensitize tubular epithelial cells to apoptosis and necrosis [129,130], or indirectly through promotion of fibrogenesis. In fact, culture of tubular cells in hypoxic conditions reduces MMP activity and increases total collagen content [131]. Also, in experimental CKD, hypoxia-inducible factor (HIF) has been shown to mediate hypoxia-induced fibrosis [132,133]. Fibrosis also affects the diseased renal vascular tree by reducing the lumen of individual vessels and peritubular capillary cross sectional area [134]. Figure 3 depicts a prototypical tubulointerstitial situation showing the most important extracellular mediators of key pathological events.

## Glomerular diseases

Glomerulopathies are renal disorders affecting glomerular structure and function. Primary glomerulopathies encompass inflammatory glomerular diseases (glomerulonephritis) and non-inflammatory glomerulopathies [135]. In addition, secondary glomerulopathies result from primary tubulointerstitial and renovascular diseases, which contribute to the progression of the damage [95]. Primary inflammatory and non-inflammatory conditions give rise to the nephritic and nephrotic syndromes, respectively [135]. Diabetes, hypertension and glomerulonephritis represent the major causes of chronic renal failure in glomerular diseases [136].

Inflammatory glomerular diseases are due to (i) systemic and renal infections; (ii) focal and segmental glomerulonephritis; (iii) glomerular basement membrane damage resulting from immune deposits in the capillary wall (lupus nephritis, membranoproliferative glomerulonephritis), accumulation of IgA complexes in the glomerulus (IgA nephropathy) and others; and (iv) vasculitic glomerulonephritis. Glomerulonephritis involves glomerular inflammation. Cellular and humoral immune responses participate in this injury, which involve circulating and in situ-formed immunocomplexes [137], and complement pathways [138], which tend to accumulate in the components of the filtration barrier and to disrupt its structure. A major consequence of glomerulonephritis is the nephritic syndrome characterized by hematuria and proteinuria (due to alterations in the glomerular filtration barrier) and by reduced glomerular filtration, oliguria and hypertension due to fluid retention [139]. Additional characteristic hallmarks of glomerulonephritis include the activation and proliferation of mesangial cells [135] and endothelial cells [140], which contribute to the fibrosis and sclerotic scar lesions commonly observed in damaged glomeruli.

Non-inflammatory glomerular diseases comprise a repertoire of metabolic and systemic diseases that chemically or mechanically damage the glomerulus, such as diabetes and hypertension, toxins and neoplasias. Non-inflammatory glomerular diseases also include idiopathic membranous nephropathy because, although it results from immune injury to the podocyte, glomerular inflammation is not conspicuous, at least initially. Diabetes is the leading cause of CKD and ESRD in developed countries, resulting in 20-40% of all patients developing ESRD [141]. Persistent hypertension is another important trigger of non-inflammatory glomerular disease, caused by pathologic remodeling of the capillary tuft as a response of an increased perfusion pressure and physical stress. Although the autoregulatory capacity of renal blood flow effectively protects the kidneys against hypertension, protection is mostly but not completely effective, and autoregulation partially fades away in a slow but progressive manner [142]. The major clinical syndrome produced by non-inflammatory glomerulopathies is the nephrotic syndrome. It presents with severe proteinuria (> 3 g/day), hypoalbuminemia, oedema, hyperlipidemia and lipiduria [139], with reduced or even normal glomerular filtration. Contrarily to the nephritic syndrome, the nephrotic syndrome courses without hematuria. Yet, it must be emphasized that even non-inflammatory glomerulopathies course with renal inflammation, which is a key mechanism of progression and an important target for therapeutics [143]. The difference with inflammatory glomerulopathies is that inflammation is secondary to the injury inflicted by the initiating cause.

### Histopathological alterations and consequences of the glomerular damage

Glomerular pathogenetic mechanisms are as diverse as types of primary glomerulopathies. Dependent on the aetiology, specific glomerular diseases course with a specific mix of renal histopathological findings or patterns, including focal and segmental sclerosis, diffuse sclerosis, mesangial, membranous or endocapillary proliferation, membranous alterations and immune deposits, crescent formations, thrombotic microangiopathy, vasculitis and others. A determined glomerular disease may evolve through different histopathological patterns. As an example, diabetic nephropathy has been recently classified in 4 types: (i) Class I, characterized by isolated glomerular basement membrane thickening and only mild, nonspecific changes by light microscopy; (ii) Class II, in which mild (IIa) or severe (IIb) mesangial expansion is observed without nodular sclerosis, or global glomerulosclerosis in more than 50% of glomeruli. (iii) Class III, when nodular sclerosis or Kimmelstiel-Wilson lesions are present in at least one glomerulus with nodular increase in mesangial matrix, without changes described in class IV; and (iv) Class IV or advanced diabetic glomerulosclerosis, characterized by the presence of more than 50% of the glomeruli with global glomerulosclerosis, and further clinical or pathologic evidence ascribing sclerosis to diabetic nephropathy [144].

**Figure 4:**
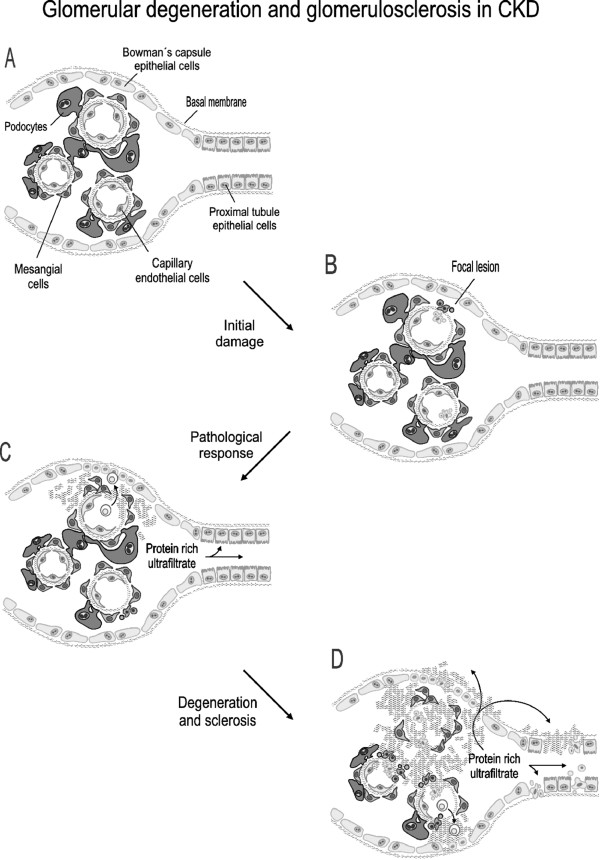
**Schematic representation of the typical pathological process of glomerular degeneration and sclerosis in glomerular diseases**. A, structure of a normal corpuscle showing the Bowman's capsule binding the glomerular capillary tuft, mainly composed of endothelial and mesangial cells, podocytes and a basal membrane. The very proximal segment of the tubule is also depicted. B, an initial insult of undetermined nature produces a focal lesion leading to podocyte loss and activation of an inflammatory response involving circulating and resident inmmune system cells. C, superseding the normal repair process, a pathological response occurs, which commonly presents with mesangial and Bowman's capsule epiyhelial cell proliferation, limphocyte extravasation and infiltration, fibrosis, and podocyte loss. The ultrafiltration membrane becomes leakier and more permeable to proteins. D, fibrosis extends damage through the corpuscle by inducing apoptosis of epithelial cells and filling the spaces left by dead cells, all of which give rise to pathways connecting the Bowman's capsule with the interstitium through with the protein rich ultrafiltrate accesses other areas of the corpuscle and the tubules and causes further damage.

In most CKDs, sooner or later the selectivity and permissivity of the glomerular filtration barrier becomes altered, and the glomerular structure collapses and leads to sclerosis and scarring, reduced glomerular flow and filtration, or even physical scission from the tubule [[145], and figure 4]. Mesangial cell proliferation and glomerulosclerosis, are also common features of most established glomerulopathies [136,146,147]. Mesangial proliferation is often considered an initial, adaptive response that eventually loses control and develops into a pathological process. Podocyte injury is another characteristic of many glomerulopathies, and a central event in proteinuric nephropathies [146,147]. Pathological podocyte involvement is mainly the consequence of (i) podocytopenia resulting from podocyte apoptosis and EMT; or (ii) foot process effacement and alterations in podocyte dynamics [146,148,149]. Podocytopenia is believed to cause or favor the adhesion of a glomerular capillary to Bowman's capsule at a podocyte deprived basement membrane point. These adhesions create gaps in the parietal epithelium that allow ectopic filtration out of Bowman's capsule into the paraglomerular, interstitial space, which may be extended over the glomerulus and may also initiate tubulointerstitial injury (150; see section 5).

Glomerular endothelial cells are also primary sites of injury resulting in glomerulopathies and CKD. They will be addressed in section 4, along with other renovascular diseases. Besides thrombotic microangiopathy, glomerulo-vascular diseases include atherosclerotic microembolia, small vessel vasculitis, diabetic nephropathy, membranoproliferative and post-infectious glomerulonephritis, lupus nephritis and the inherited disease familial hemolytic uremic syndrome. In addition, the hemodynamic damage is an important component of glomerulosclerosis and progressive glomerular injury in most forms of CKD. Hyperfiltration, glomerular hypertension, glomerular distention and inflammation occurring after the initial insult cause diverse glomerular alterations that activate, and even damage, mesangial and endothelial cells [[151]; see also section 5].

Glomerular ECM deposition evolves in patients with glomerulonephritis as the disease progresses [152]. As in normal kidneys, no interstitial collagen I and III are detected in patients with mild glomerulonephritic damage [152]. Progressive renal damage correlates with increasing presence of collagen IV and VI, laminin and fibronectin in the mesangium. Finally, in later stages of glomerulonephritis, the amount of collagen IV, laminin and fibronectin gradually decreases, while focal expression of collagen I and III increases. Glomerular cell apoptosis also occurs in parallel to sclerosis, and ECM progressively scars the spaces left by dead cells [153].

Inflammation plays a pivotal role in the progression of many, if not all, forms of CKD. In the glomerulus, inflammation exerts different effects that amplify the damage and directly contribute to the reduction in glomerular filtration (see section 3.2.). Initially, inflammation is probably activated as a repair mechanism upon cellular and tissue injury. However, undetermined pathological circumstances skew persistent inflammation into a vicious circle of destruction and progression. In fact, inflammation activates many renal cell types to produce cytokines, which directly damage renal cells and intensify inflammation.

### Cells and molecular mediators involved

Mesangial cells are contractile glomerular pericytes that play a major role in the regulation of renal blood flow and GFR. They also have a pivotal participation in the genesis of chronic glomerular diseases. Mesangial cell proliferation is a common feature during the initial phase of many chronic glomerular diseases, including IgA nephropathy, membranoproliferative glomerulonephritis, lupus nephritis, and diabetic nephropathy [154]. Numerous experimental models of glomerular damage have reported that proliferation of mesangial cells frequently precedes and is associated with ECM deposition in the mesangium and, therefore, to fibrosis and glomerulosclerosis. In fact, reduction of mesangial cell proliferation in glomerular disease models ameliorates ECM deposition, fibrosis and glomerulosclerosis [154]. Thus, proliferating mesangial cells are considered to be a central source of ECM production in both focal and diffuse glomerulosclerosis [155,156].

The fibrotic mechanism of renal damage in glomerulopathies represents a final common pathway with the initial glomerular insult starting a cascade of events that include an early inflammatory phase followed by a fibrogenic response in the glomerular and the tubulointerstitial compartments of the kidneys [93]. Several cytokines, growth factors and complement proteins, through the activation of nuclear factor-κB (NF-κB)-related pathways, initiate the damage stimulating the mesangial cells to release chemotactic factors [157]. As previously reported, angiotensin II is one of the main effectors implicated in resident cell activation in pathological kidney [126]. Infusion of angiotensin II induces a marked renal damage in glomeruli, tubulointerstitium and vascular system, associated with cell proliferation, leukocyte infiltration, interstitial fibrosis and modulation of mesangial cell phenotype [158]. In the short-term, angiotensin II acting on mesangial cells induces an increase of cytosolic calcium and inositol phosphate, prostaglandin synthesis and cellular contraction and long-term alterations such as proliferation, hypertrophy and ECM production [159]. These effects are mediated by autocrine factors released upon angiotensin II action, such as TGF-β1 [86,136,160]. TGF-β induces mesangial cell proliferation directly and through the concourse of PDGF [161]. PDGF appears to be an important mediator of mesangial proliferation, and HGF counteracts PDGF actions [162]. Several pathogenic molecules have been additionally related to the development of glomerulosclerosis, including endothelin [163] and reactive oxygen species [164] that have also been implicated in angiotensin II-induced hypertrophy of mesangial cells [165].

**Figure 5:**
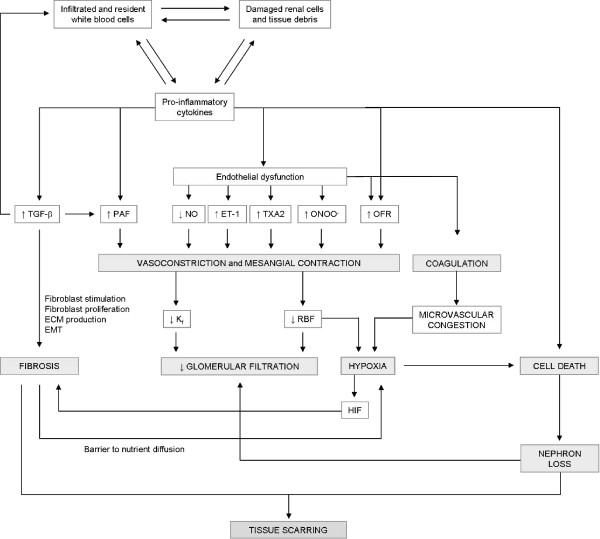
**Glomerular effects of inflammation**. ET-1, endothelin 1. HIF, hypoxia inducible factor. K_f_, ultrafiltration coefficient. OFR, oxygen free radicals. PAF, platelet activating factor. RBF, renal blood flow. TGF-β, tumor growth factor beta. TXA2, thromboxane A2.

Resident glomerular cells and circulating inflammatory cells, including neutrophils, platelets and macrophages mediate inflammatory responses leading to glomerular lesions [135,166,167]. Recruited inflammatory cells amplify the fibrotic and proliferative response of mesangial cells [168], and also the expression of the EMT marker α-SMA [169], the production of ECM components [155,170], and exacerbate cytokine and growth factor release [171]. As explained for tubulointerstitial diseases (sections 2.1. thru 2.3.), pro-inflammatory cytokines, including TNF-α, IL-1 and other interleukins, interferon gamma, tweak and others, are known to be involved in paracrine actions resulting in (figure 5):

(i)Direct cell injury and death [172,173].

(ii)Stimulation of TGF-β production by renal cells [174] and fibrosis [175,176].

(iii)Renal vasoconstriction that diminishes renal blood flow with two consequences: on the one hand it diminishes glomerular filtration, and on the other, it may lead to oxygen deficit and hypoxia in determined circumstances. Hypoxia sensitizes cells to cell death and activates the release of HIF, which promotes fibrosis [131-133]. Besides, hypoxia limits the cell's ATP reserve and thus it may change the cell death phenotype to necrosis [177], which in turn further activates the immune response. Vasoconstriction might be the result of endothelial dysfunction and oxidative stress [178-180], and also of release of contracting factors such as endothelin-1 and platelet activating factor (PAF) by endothelial and mesangial cells, and podocytes [181-184].

(iv)Microvascular congestion resulting from endothelial dysfunction and aberrant coagulation, which contributes to hypoxia [185,186].

(v)Mesangial contraction [181-184], causing the ultrafiltration coefficient (K_f_) and glomerular filtration to decrease [187].

Proliferating parietal epithelial cells (PECs) of Bowman's capsule have been involved in the development of FSGS, and in extracapillary proliferation. Long considered passive bystanders in CKD, in recent years several studies have shown that PECs proliferate and produce ECM components contributing to fibrosis, adhesions of glomerular capillary to Bowman's capsule [188,189], and glomerular collapse, in different glomerular diseases. In addition, PECs can become activated and express many growth factors, chemokines, cytokines, and their receptors [reviewed in [190]].

Finally, podocytes have progressively gained central attention in glomerulopathies and are considered to have a central role in the pathological process, as a result of both genetic and acquired alterations. Loss of podocytes, which lack the ability of postnatal proliferation, has been implicated in the progression of glomerular diseases to glomerulosclerosis [191]. Podocytes are specialized pericytes placed around the glomerular capillaries, which contribute to the special characteristics of the glomerular filtration barrier [148,192]. Human acquired proteinuric glomerulopathies, such as diabetic nephropathy, minimal-change nephrotic syndrome (MCNS), FSGS, and membranous nephropathy (MN), commonly exhibit foot process effacement of podocytes and loss of slit diaphragms in electron microscopy; these glomerulopathies therefore are considered as podocyte injury diseases (podocytopathies) [148,193]. Several experimental models, such as rat puromycin aminonucleoside (PAN) nephropathy and mouse adriamycin (ADR) nephropathy that develop massive proteinuria resembling human minimal change disease, have provided insights into the cellular and intracellular mechanisms of podocyte injury disease.

Podocyte dysfunction leads to progressive renal insufficiency. First, podocyte damage causes proteinuria. Sustained proteinuria gives rise to tubulointerstitial injury, eventually leading to renal failure [194]. Second, podocyte injury impairs mesangial structure and function. In anti-Thy-1 glomerulonephritis, the induction of minor podocyte injury with PAN pretreatment results in an irreversible mesangial alteration [195]. In addition, cysteine-rich protein 61 (Cyr61), a potent angiogenic protein that belongs to the CCN family of matrix-associated secreted protein family, is expressed in podocytes and upregulated in anti-Thy-1 glomerulonephritis [196]. Cyr61 inhibits mesangial cell migration, suggesting that Cyr61 may play a modulatory role in limiting mesangial activation. Thus, podocytes may secrete various humoral factors that regulate mesangial structure and function, and their reduction could result in impaired mesangial function, mesangial proliferation and matrix expansion. For example, angiotensin II and high glucose exposure increase podocyte production of TGF-β1 [197] and VEGF [198], both of which are known to affect mesangial cells [199]. Third, podocyte loss or detachment from the glomerular basement membrane leads to glomerulosclerosis [200]. In human diabetic nephropathy and IgA nephropathy, decreased podocyte number correlates significantly with poor prognosis [201,202]. These data suggest that podocyte injury is critical not only in podocyte-specific diseases such as MCNS and FSGS but also in podocyte-nonspecific diseases such as IgA and diabetic nephropathy.

## Renovascular diseases

Renovascular diseases comprise a group of progressive conditions involving renal dysfunction and renal damage derived from the narrowing or blockage of the renal blood vessels. According to the U.S. Renal Data System [203], about one third of all ESRD cases were related to renovascular diseases. Renovascular diseases usually appear as microangiopathies, although renal artery occlusion, renal vein thrombosis, and renal atheroembolism are also potential causes. The term is most often used to describe diseases affecting the renal arteries, because blockage of the renal veins is not very common. Renovascular alterations affect the main renal arteries and their branches (stenosis) or microvessels (thromboembolic microangiopathy) and lead to CKD. Atherosclerosis induces 70-90% of cases of renal stenosis and is the predominant lesion detected in patients >50 years of age [204,205], whereas most remaining cases are caused by fibromuscular dysplasia. The latter is a group of idiopathic fibrotic conditions affecting especially the media, but also the intima and the adventitial layers of small vessels, which is more frequent in middle-aged women. Unusual causes of stenosis are external pressure (e.g. exerted by a tumor), partial occlusion at the suture level after renal transplant, as well as nephroangiosclerosis (hypertensive injury), diabetic nephropathy (in small vessels), renal thromboembolic disease, atheroembolic renal disease, aortorenal dissection, renal artery vasculitis, trauma, neurofibromatosis, thromboangiitis obliterans and scleroderma [206,207]. CKD is a probable outcome, although stenotic hypoperfusion is not synonymous with renal disease. Surprisingly, stenosis caused by fibromuscular dysplasia rarely provokes renal damage, despite inducing intrarenal hemodynamic changes and activating pressor mechanisms as well. On the contrary, atherosclerotic stenosis more often leads to CKD. Even moderate stenosis can (more rarely) give rise to CKD. The likelihood of developing CKD associated with atherosclerotic stenosis escalates with the severity and persistence of the occlusion and with the presence of comorbid factors [208].

As explained in the next paragraphs, renovascular diseases may alter renal function and structure directly through (i) atherosclerosis-initiated renal oxidative stress, endothelial dysfunction and inflammation leading to fibrosis and reduced filtration; (ii) creating hypoperfusion and ischemic scenarios compromising renal blood flow, and tubular and glomerular function; and (iii) indirectly, through the onset of hypertension.

### Atherosclerosis and renal injury

**Figure 6:**
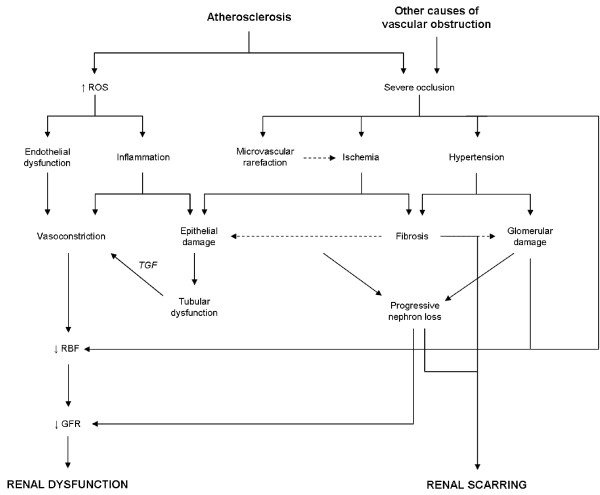
**Initiating mechanisms in renovascular nephropathies**. GFR, glomerular filtration rate. RBF, renal blood flow. ROS, reactive oxygen species. TGF, tubulo-glomerular feedback

Atherosclerosis of renal vessels has two main effects leading independently and cooperatively to renal dysfunction. On the one hand, atherosclerotic vessels have an increased production of ROS that cause oxidative stress. Oxidative stress has two main consequences: (i) endothelial dysfunction, and (ii) inflammation. On the other hand, large atherosclerotic formations may reduce renal blood flow (in the whole kidney or in specific areas) over the auto-regulatory window, and sufficiently to reduce glomerular filtration [figure 6; [209,210]]. Even in the absence of an important obstruction, endothelial dysfunction and inflammation can cause glomerular filtration to decrease. Endothelial dysfunction causes vasoconstriction and reduced renal blood flow leading to reduced filtration. Inflammation induces tubular and glomerular cell activation and the production of vasoactive molecules, such as platelet activating factor (PAF), endothelin-1 and RAS activation [143]. These mediators induce (i) vasoconstriction and mesangial contraction (which reduces the ultrafiltration coefficient, K_f_) leading to the reduction of glomerular filtration; and, in some circumstances (ii) cell death contributing to nephron loss.

Increased production of ROS in pathological situations such as hypertension and atherosclerosis is frequently mediated by activation of the renin-angiotensin system and NAD(P)H oxidase [211-213]. As Chade et al. [214] showed that systemic plasma renin activity was not elevated in an in vivo experimental model of renovascular disease, the intrarenal renin-angiotensin system seems to be activated within the stenotic kidney. The angiotensin II-induced ROS generation through activation of NAD(P)H oxidase seems to involve a feed-forward mechanism inducing a prolonged production of ROS [211]. Chronic effects of oxidative stress play a relevant role in the pathogenesis of renal injury in renovascular disease [214], and oxidative stress clearly contributes to renovascular-induced hypertension [215]. ROS may induce vasoconstriction and modulate renal microvascular function [216], contributing to the enhanced renal vascular tone and sensitivity induced by other vasoconstrictors such as angiotensin II and endothelin-1. Furthermore, superoxide anion and nitric oxide (NO) may also react with each other, which decreases NO availability and impairs intrarenal vascular and glomerular function due to the formation of peroxynitrite [213,216]. Finally, antioxidants have shown to prevent the renal damage and dysfunction induced by renal artery obstruction and atherosclerosis [214]. All these facts suggest that increased oxidative stress is involved, at least partially, in the impaired endothelium-dependent vasodilatation observed in patients with renovascular hypertension.

### Renal injury due to hypoperfusion and ischemia

Severe occlusions decreasing over a 60% of renal flow, lead to a reduction of renal perfusion pressure under the autoregulatory range (< 70-85 mmHg). Renal hypoperfusion appears only when renal perfusion pressure falls below the autoregulatory range, and thus renal blood flow declines. It is estimated that a 70-80% of the luminal area of the renal artery must be occluded for hypoperfusion to occur, which is termed "critical stenosis" [217]. This condition induces a generalized tissue hypoperfusion (sometimes referred to as ischemia) and excretory dysfunction, which may evolve to fibrosis (frequently to secondary FSGS) and CKD. Localized or spread thromboembolic microangiopathy may also cause focal or generalized true ischemic scenarios, which may be the consequence of systemic atherosclerotic disease, or may be indirectly potentiated by it through main renal artery atherosclerotic stenosis. Still, a severe diminution of renal blood flow does not necessarily cause an injuring ischemia, but it may merely lead to a reversible, hibernating-like functional state and in some cases to renal damage [208]. It must be born in mind that just a mere 10% of total oxygen passing through the kidney is used for its metabolic needs [218]. In this situation, pressor mechanisms become invariably activated which raise systemic blood pressure and, consequently, renal perfusion pressure to achieve water and electrolyte balance (see 4.3.). Hypertension aggravates the renal stenosis outcome [219]. In fact, a complex relationship has been described among renal artery stenosis, hypertension and CKD [220].

Severe renal hypoperfusion leads to microvascular rarefaction (MR) and deficient vascular endothelium growth factor (VEGF) production and focal or spread ischemia [221]. MR seems to play a significant role in renovascular disease, because exogenous administration of VEGF prevents MV and renal dysfunction [221]. Ischemia also is recognized as a strong injuring and fibrogenic stimulus, but the mechanisms leading to CKD are poorly understood [208]. HIF, which is a pro-angiogenic and protective mediator in vivo released by ischemic cells, has been demonstrated to promote renal fibrosis in chronic pathological circumstances [222]. Finally, renal hypoperfusion has been linked to tubular injury [223]. Decreased O_2 _and glucose supply limit ATP production, which leads or predisposes cells to dying [224-226]. Hypoxia also activates inducible nitric oxide synthase (iNOS) expression, which produces oxidative stress, inhibits ATP synthesis and activates apoptosis [227].

### Hypertensive injury

Hypertension is a prospective inducer of renal damage in stenotic kidneys [228]. Hypertensive nephropathy is a glomerulopathy initiated by the increase in intraglomeular pressure, which activates and damages glomerular cells, including mesangial and epithelial cells and podocytes. These cells produce proinflammatory and vasoactive mediators that contribute to cell damage and fibrosis, reduce renal blood flow, Kf, and glomerular filtration (as described in general for glomerulopathies in section 3, and specifically in 143; and depicted in figure 6). Initially, hypertension-induced stress activates the local RAS at the glomerular level. As in many other cardiovascular pathological situations, local RAS has been decisively implicated in tissue alteration and remodelling. Renal TGF-β, NF-κB and other cytokines are upregulated in a model of hypercholesterolemic renovascular CKD [229], and also in a model of aortic coarctation between both renal arteries, which pathologically resembles unilateral stenosis [230,231]. They might mediate the inflammatory, fibrotic and apoptotic events, as described generally for glomerular and tubular diseases [208].

**Figure 7:**
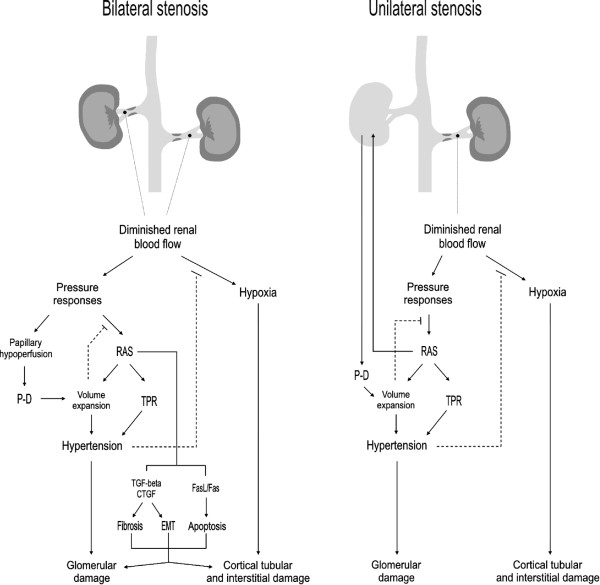
**Pathophysiological events characteristic of the chronic phase of bilateral and unilateral stenotic renal disease**. In both cases, the hypoxia created by a substantially diminished renal blood flow and the hypertensive response are the dominant damaging mechanisms (see text). RAS, renin-angiotensin system. TPR, total peripheral resistance. P-D, pressure diuresis. EMT, epithelial to mesenchymal transition.

Renal artery stenosis may affect one kidney or both, which induces different pathological scenarios [figure 7]. The case of a stenotic solitary kidney (as in unilaterally nephrectomized or transplanted patients) is similar to that of bilateral stenosis. In all cases, RAS plays a central role in initiating compensatory responses that involve systemic pressure rise [232]. In bilateral stenosis and solitary stenosed kidneys the reduced perfusion pressure induces a rapid release of renin that results in an increased production of renal and systemic angiotensin II. This, in turn, provokes a strong renal and systemic vasoconstriction, and sodium and water tubular resorption that swiftly induce hypertension. Importantly, after a few days, renin release by the stenotic kidney returns to normal values, and hypertension becomes dependent on extracellular (and blood) volume expansion and independent from the RAS. Angiotensin converting enzyme inhibitors (ACEIs) no longer affect blood pressure, despite being capable of preventing its onset. If sodium and water depletion is induced, hypertension becomes newly renin-dependent [208,232]. RAS-mediated blood pressure control is considered a medium term mechanism. After that, pressure-natriuresis supersedes other control mechanisms and even inactivates RAS-mediated control [142], by turning down renin release [208,232].

In unilateral stenosis, the obstructed kidney responds as in bilateral stenosis with renin release, angiotensin II production and hypertension. In unilateral stenosis, maintenance of hypertension is dependent on a constantly activated RAS. High levels of circulating and renal angiotensin II become increased [233], which probably reset the pressure-natriuresis-diuresis mechanism in the non stenotic kidney to higher levels of pressure, so that water and electrolyte balance is achieved at the new pressure. In fact, RAS blockers (e.g. angiotensin converting enzyme inhibitors, ACEIs) inhibit both the appearance and maintenance of hypertension in this model [207]. It is noteworthy that angiotensin II is capable of sustaining hypertension in the long term, as demonstrated by the experimental rat hypertension model induced by constant administration of angiotensin II [234]. In unilateral stenosis (and associated experimental models, e.g. the Goldblatt experimental model of unilateral stenosis, "two-kidney, one-clip" -2K1C-, and the aortic coarctation between the renal arteries) the non stenotic kidney also undergoes structural alterations [230,231], probably as a consequence of the developed hypertension, or as a result of the systemic or local humoral alterations switched as a compensatory response. In fact, TGF-β expression is upregulated as well in the contralateral kidney by 3-5 weeks after stenosis in 2K1C [235].

## Merging mechanisms of progression

**Figure 8:**
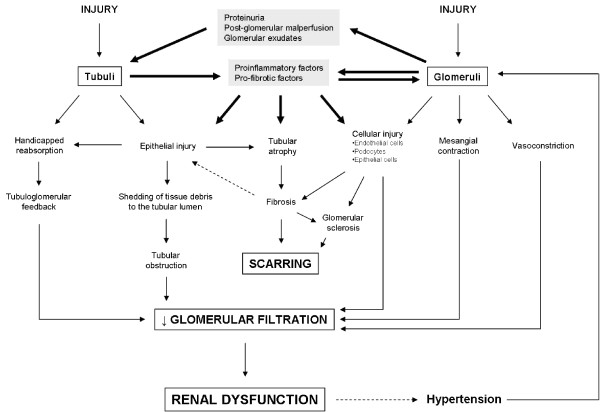
Pathological events linking glomerular and tubular injury, which lead to a progressively commoner phenotype as CKD progresses.

Irrespective of the cause, CKD pathogenesis is characterized by a progressive loss of renal function, and an excessive deposition of extracellular matrix in the glomeruli and tubular interstitium [236]. CKD progression is associated with the appearance of an increasingly commoner, fibrotic phenotype, where it is difficult to determine the origin of the disease except for very subtle morphological characteristics only available through the pathological examination of renal biopsies. This is because tubulointerstitial diseases ultimately induce glomerular lesions, and glomerular diseases eventually cause tubulointerstitial damage. In both cases, the result is a progressive nephron deletion and substitution for scar-like tissue, which increasingly reduce glomerular filtration and thus handicap renal excretory function. Importantly, the degree of the renal lesion and the risk of progression closely correlate with the extent of tubulointerstitial fibrosis, regardless of etiology [69]. This suggests that, at least initially, damaged glomeruli have less impact in renal excretory function than damaged tubuli. Damaged and sclerotic glomeruli may retain an undetermined degree of filtration function which, due to renal reserve, may cause a lower impact in the overall renal function. However, a mild dysfunction in tubular reabsorption may lead to a dramatic fall in glomerular filtration through the activation of the tubuloglomerular feedback retrocontrol, in order to preserve hydroelectrolytic balance [237]. Furthermore, damaged tubuli may get partially or totally obstructed by tissue debris resulting from epithelial destruction, which reduces or stops filtration [figure 8]. Damaged tubuli produce a number of pro-fibrotic and pro-inflammatory factors that, in pathological circumstances, may also alter glomerular function and damage glomeruli in a paracrine manner (see table 2 and [143]).

Mechanisms traditionally suggested to connect primary glomerulopathies with the subsequent pathological recruitment of the tubulointerstitial space are [238]: (i) an increased reabsorption of proteins in the proximal tubules, resulting from glomerular hyperfiltration associated with glomerular damage. An increased tubular reabsorbtion of proteins activates the production of cytokines by tubular cells, which, in turn, promotes the infiltration of immune cells and the activation of an immune-inflammatory response (238; and see the section "Historical view" in 2). Abnormally filtered bioactive macromolecules interact with proximal tubular epithelial cells, activating signalling pathways that include NFkB [239,240]. The megalin-cubilin complex mediates the uptake of several proteins, including albumin, into proximal tubular epithelial cells. Megalin might also initiate or participate in intracellular signalling linking abnormal albuminuria with proinflammatory and profibrotic signaling [240]. The neonatal Fc receptor and CD36 could also play a role. Furthermore, addition of albumin or transferrin to tubule cells reduces their ability to bind factor H and counteract complement activation [241]. Albumin can also be a source of potentially antigenic peptides upon processing by renal dendritic cells [242]. Indeed, proteinuria is not only a marker of disease, but also an effector of nephropathy. Proteinuria correlates with disease progression, and pharmacological prevention of proteinuria also correlates with progression slowing [2]; (ii) direct encroachment of extracapillary lesions from the glomerulus to the tubule [150]; (iii) recurring acute glomerular insults (as by toxics, metals, drugs, infections, etc.) which perpetuate the production of growth factors and chemokines involved in tubular damage [238]; (iv) postglomerular malperfusion derived from the degradation, collapse or narrowing of glomerular capillaries, resulting in tubular hypoxia [238]; (v) formation of paraglomerular exudates containing profibrotic factors, ECM, basement membrane material and tissue debris from epithelial cells and podocytes, which reach the tubular structures through interstitial routes and initiate an injury process leading to tubulointerstitial fibrosis and tubule degeneration that, in some instances, may lead to the physical separation of the glomerulus and the tubule, and the formation of a glomerular cyst [238]. Sclerotic nuclei begin at glomerular adhesions formed by a glomerular capillary to Bowman's capsule at a podocyte deprived basement membrane point, which lead to the formation of a paraglomerular space (PGS). PGS contains ectopic filtrate and capillary tuft debris. The PGS content is proposed to play a significant role in the initiation of damage and in the connection of glomerular and tubular diseases. It must be pointed out that increasing evidence suggests that even in traditionally considered glomerulopathies, such as diabetic nephropathy, some degree of tubular damage occurs before the first evidence of glomerular injury can be detected [243-247]. This may eventually force us to reshape our conceptual separation of glomerular and tubular diseases into a more integrative view [245].

Regardless of cause, as a consequence of the increasing renal dysfunction, compensatory responses are activated, which may also engage in the progressive pathological vortex. These responses include hypertension and peripheral or renal sympathetic hyperactivity [248], which are commonly observed in CKD patients. Indeed, baroreceptor-mediated renal sympathetic hyperactivity has been recently linked to the inception and maintenance of hypertension [142]. Figure 8 compiles the pathological mechanisms connecting tubular and glomerular damage, which set the basis of a common renal pattern of disease during the progression of CKD.

## Conclusions, clinical implications and perspectives

This review summarizes the key pathophysiological events of CKDs compromising renal excretory function, at the organism, tissue, cell and molecular levels. CKDs may be originated in the glomeruli, in the tubuli or in the renal vessels. Most of the diseases in each of these groups have specific, but also common pathophysiological characteristics resulting from increasingly understood mechanisms of action. Moreover, all these diseases, regardless of aetiology, eventually affect all parts of the nephron and enter an irreversible course that may compromise the patient's life. In addition, as the disease progresses, a more uniform pathophysiological pattern installs characterized by increasing fibrosis, inflammation, nephron loss and parenchymal scarring. Present treatments of CKD are only reasonably effective at slowing progression. They are installed substantially after irreversibility ensues, mostly because clear pathological signs only arise after over 50% of the nephrons are functionally nulled. In these conditions, the earliest possible diagnosis is critical for prognosis. Moreover, the identification of new biomarkers and technologies to move progressively earlier the moment of diagnosis is an active area of research.

The follow-up of CKD patients shows that the overall death rate increases as kidney function decreases, and the mortality in patients with ESRD remains 10-20 times higher than that in the general population. At present there is no cure for CKD; the natural course of the disease is to progress towards ESRD and death, unless dialysis or transplant is implemented. The focus in recent years has thus shifted to optimizing the care of these patients during the phase of CKD, and to slow progression with the aim of avoiding the necessity of renal replacement therapy during the patient's lifespan. In many cases, it is possible to slow the progression of CKD to ESRD if kidney disease is diagnosed and treated in its earlier stages, mainly with renin-angiotensin system blockers, although other drugs are under development based on known mechanisms of progression [143,249]. Thus, early CKD identification has potentially enormous socioeconomic and medical benefits. Still, the development of earlier diagnostic tools and better drugs for preventing and, ideally, reversing renal damage and restoring renal function needs a better knowledge of pathophysiological mechanisms of CKD genesis and progression. In this sense, reversal of CKD in the clinical setting is still an unmet goal. However, promising results have been obtained in some studies with experimental models of renal fibrosis, for instance using BMP-7 as a therapeutic agent [250,251].

Yet, a valuable and potentially useful piece of knowledge for the clinical handling of CKD is still in the horizon; namely understanding how and why an initial or persistent insult to the kidney is not repaired but, on the contrary, leads to an irreversible scenario of self destruction, which even becomes independent from the cause. This no-return point in the fate of injured kidneys probably holds the key to a conceptual therapeutic drift from slowing progression towards regression and, along with a sufficiently early diagnosis, prevention entering the vicious circle of deterioration. As it has been suggested that an imbalance of pro-fibrotic and anti-fibrotic cytokines is in the core of the no-return point [110], it would be helpful to focus research efforts on this key aspect of CKD, as a way to gain true control over this disease.

## Declaration of competing interests

The authors declare that they have no competing interests.

## Authors' contributions

JML-N drafted the manuscript and contributed with specific information and critical analysis through the manuscript. ABR-P provided most of the information on tubulointerstitial diseases. AO introduced the clinical scope to the manuscript and specific aspects of sections 1 and 2.3. CM-S incorporated a part of the information in sections 3 and 4, provided specific pieces of information through the manuscript and critically helped with the draft. FJL-H delineated and wrote most of the manuscript, composed the figures and integrated the information into sections 5 and 6. All authors read and approved the final manuscript.
